# How absolute is zero? An evaluation of historical and current definitions of malaria elimination

**DOI:** 10.1186/1475-2875-9-213

**Published:** 2010-07-22

**Authors:** Justin M Cohen, Bruno Moonen, Robert W Snow, David L Smith

**Affiliations:** 1 Clinton Health Access Initiative, 383 Dorchester Ave Suite 400, Boston MA 02127, USA; 2 Kenya Medical Research Institute/Wellcome Trust Collaborative Programme, Centre for Geographic Medicine, Nairobi & Kilifi, Kenya; 3 Centre for Tropical Medicine, Nuffield Department of Clinical Medicine, CCVTM, University of Oxford, UK; 4 Emerging Pathogens Institute, University of Florida, Gainesville, Florida, 32610-0009, USA; 5 Department of Biology, University of Florida, Gainesville, Florida, 32611, USA

## Abstract

Decisions to eliminate malaria from all or part of a country involve a complex set of factors, and this complexity is compounded by ambiguity surrounding some of the key terminology, most notably "control" and "elimination." It is impossible to forecast resource and operational requirements accurately if endpoints have not been defined clearly, yet even during the Global Malaria Eradication Program, debate raged over the precise definition of "eradication." Analogous deliberations regarding the meaning of "elimination" and "control" are basically nonexistent today despite these terms' core importance to programme planning. To advance the contemporary debate about these issues, this paper presents a historical review of commonly used terms, including control, elimination, and eradication, to help contextualize current understanding of these concepts. The review has been supported by analysis of the underlying mathematical concepts on which these definitions are based through simple branching process models that describe the proliferation of malaria cases following importation. Through this analysis, the importance of pragmatic definitions that are useful for providing malaria control and elimination programmes with a practical set of strategic milestones is emphasized, and it is argued that current conceptions of elimination in particular fail to achieve these requirements. To provide all countries with precise targets, new conceptual definitions are suggested to more precisely describe the old goals of "control" - here more exactly named "controlled low-endemic malaria" - and "elimination." Additionally, it is argued that a third state, called "controlled non-endemic malaria," is required to describe the epidemiological condition in which endemic transmission has been interrupted, but malaria resulting from onwards transmission from imported infections continues to occur at a sufficiently high level that elimination has not been achieved. Finally, guidelines are discussed for deriving the separate operational definitions and metrics that will be required to make these concepts relevant, measurable, and achievable for a particular environment.

## Background

Since the goal of global malaria eradication was resurrected in 2007 [1], discussions of the proper aims of national malaria programs have been revitalized [2-4]. Today, global malaria eradication - or the permanent reduction to zero of the worldwide incidence of infection [5] - is considered infeasible with currently available tools [6], but 39 countries are contemplating elimination [7], generally defined in the same way as eradication but on a country or regional scale and thus necessitating continued measures to prevent reestablishment of transmission [5].

Decisions to eliminate malaria involve a complex set of factors [8], and measuring malaria itself involves a number of uncertainties [9,10]. This complexity will be compounded by any ambiguity surrounding terminology. Despite the need for precise definitions [11], there has never existed universal agreement over the meaning of many terms of basic relevance to malaria programs [9]. Even during the Global Malaria Eradication Program (GMEP), debate raged over what, exactly, "eradication" meant. At one pole, Cockburn argued that "*so long as a single member of the [pathogen] species survives, then eradication has not been accomplished*" [12], a state that today is called "extinction" [13]. At the other, it was posited that "*the aim of eradication of an infectious disease is its reduction to a level at which it ceases to constitute an important public health problem*" [14], a definition closely aligned with what recently has been called "control" [3]. In between these extremes, eradication was defined as the "*continued absence of transmission within a specified area*," [15] including "*the elimination of the reservoir of infective cases*" [16], an explanation more in line with the current concept of "elimination" [13].

Debates over the precise definitions of terms like "eradication," "elimination," and "control," are much more than semantic arguments. International donor agencies need measurable markers of progress to justify the billions of dollars being spent on malaria interventions, and national malaria programmes must formulate strategies and goals based on clearly defined terms. To advance the contemporary debate about these issues, this paper provides a historical review of commonly used terms, including control, elimination, and eradication, to help clarify their conceptual definitions and operationalized interpretations. The review has been supported by an analysis of the underlying mathematical concepts on which these definitions are based through simple branching process models that describe the proliferation of malaria cases following importation. To provide all countries with clear targets, new conceptual definitions are suggested to more precisely describe the old goals of "control" - here more exactly named "controlled low-endemic malaria" - and "elimination." In addition, it is argued that a third state, called "controlled non-endemic malaria," is required to describe the epidemiological condition in which endemic transmission has been interrupted, but malaria resulting from onwards transmission from imported infections continues to occur at a sufficiently high level that elimination has not been achieved. Guidelines are discussed for deriving the separate operational definitions and metrics that will be required to make these concepts relevant, measurable, and achievable for a particular environment.

### Historical review of eradication, elimination, and control

In 1981, Yuketiel wrote, "*Part of the controversy regarding the question 'control or eradication?' stems from the lack of a common, uniform concept of the meaning of the term 'eradication'*" [17]. Today, the controversy over control or elimination of malaria continues to be clouded by similarly vague terminology. For example, the Carter Center's International Task Force for Disease Eradication [18] stated that elimination can refer either to "*cessation of transmission of a disease in a single country, continent, or other limited geographic area*," or "*control of a disease or its manifestations to a level that it is no longer considered a public health problem, as an arbitrarily defined qualitative...or quantitative...level of disease control*" [19]. These dissimilar meanings have enormously different operational implications, yet most modern malaria definitions have focused on the first while ignoring the second [2,6,7,11]. The lack of contemporary debate regarding the meaning of elimination contrasts sharply with historical argument over the definition of eradication.

The clear decision of the GMEP to leave most of Africa out of "global eradication" is at odds with a conception of eradication as the worldwide reduction to zero transmission. The 5^th ^WHO Expert Committee Report, which laid out the new eradication strategy in 1956, openly stated that "*the prolonged period of the transmission season and the extremely high degree of malaria endemicity in the region" *combined with weak infrastructure "*are likely to form an effective barrier to a large-scale eradication programme*" [20]. Six years earlier, at a conference in Kampala, a recommendation was made "*to governments responsible for the administration of African territories that malaria should be controlled by modern methods as soon as feasible, whatever the degree of endemicity, and without awaiting the outcome of further experiments*" [21]. Yet by 1964, GMEP activities covered only approximately 3.2% of the populations at risk in Africa, and most of these programmes were concentrated at the margins of malaria's geographical range [22]. There is an enormous discrepancy between the recommendation of the Kampala conference, the small-scale of operations of GMEP programmes in sub-Saharan Africa, and GMEP's mission of global eradication [23].

Part of this discrepancy existed because, historically, "eradication" - derived from the Latin *ex *and *radix*, meaning "to tear out by the roots" - had no clear geographic bound. As Soper, one of the principal proponents of a global eradication campaign, explained in 1962, "*The objective of eradication is completely to eliminate the possibility of the occurrence of a given disease, even in the absence of all preventive measures. This definition, modified by the phrase 'unless reintroduction occurs,' applies also to local area, state, national, and regional eradication*" [24]. Contemporary perception that GMEP was a "failure" comes in part by comparison with the consensus modern definition that eradication can only be global [25,26].

"Regional eradication," however, was always a controversial concept. Soper, in a discussion of tuberculosis eradication, argued that it was implausible in practice despite his allowance that it existed in theory: "*In control, one may plan on a small local scale, for limited areas; in eradication, one must plan for a program of sufficient scope to minimize, from the beginning, the threat of reinfection from the periphery; in eradication there is no stopping point, no rest period. Eradication must continuously expand at the periphery until all points from which reinfection may occur have been cleared*" [24]. Regional eradication was thus only possible at the widest geographic scales. Similarly, Cockburn called regional eradication of infectious disease a "*basically unstable situation, because at any time the infection may be reintroduced by carriers or vectors from the outside*" [12]. And if control measures had to be kept in place to keep this from happening, he argued, then the programme must be one of control and not eradication at all.

Fenner describes the inherent conflict in even allowing for the possibility of local or national eradication, pointing out that since it "*requires that there is no possibility of reintroducing the pathogen from another geographic area*," and since that possibility will in most cases be impossible, "*'regional eradication' is an oxymoron because of the ever-present risk of importation of the pathogen and the continuing need for control measures*" [27].

He summarized in 1988:

*"The question of how large a specified area must be in order to apply usefully the term eradication has frequently been a contentious issue. Measles illustrates the quandary as to what the lower limits should be. The eradication of measles in a household or district in a city means little, since transmission periodically ceases in such small areas without the application of control measures, and reinfection regularly occurs. But should one speak of eradication of measles from a state or province, for example, or should the notion apply only to a continent or even larger area? Views differ on this question but most epidemiologists now prefer to use the term eradication only when the area covered is sufficiently large and geographically delimited and the characteristics of the disease or vector are such that reinfection or reinfestation is unlikely." *[28]

Fenner's notion about the futility of "regional eradication" for measles may not apply to malaria. In fact, 24 countries that "eradicated" malaria during GMEP remain malaria-free today; most of those were either islands or shared a border with another country that succeeded in interrupting malaria [29]. It is important to note, however, that even countries where regional eradication was successful continue to see occasional outbreaks of transmission as a result of importation of infections [30,31]. An essential difference between malaria and measles is that malaria transmission requires the presence of a vector and environmental conditions that support transmission. Permanent regional reductions were indeed possible, at least in certain regions where transmission was naturally amenable to control.

In other places, however, particularly those not located on islands or areas of very low intrinsic transmission potential, the instability of "regional eradication" required a different goal. The concept of "elimination" eventually was accepted as an interim state for those situations where sufficient risk of importation existed that control measures could not be relaxed without reestablishment of transmission [27] (Table 1). The term comes from the Latin roots *ex *and *limen*, meaning "beyond a threshold." Payne appears to have been among the first to suggest a discrete meaning for this term as compared to eradication in 1963, although the usage does not appear to have caught on for a further two decades. "*Since a threshold is involved*," he wrote, "*This is not a final process and the threshold specified may vary from disease to disease...Alternatively the threshold may be the boundaries of a defined geographic area*" [32].

**Table 1:** Definitions of elimination and related concepts as they have changed over time

Year	Definition of "elimination"	Source
**1961**	"Regional eradication" implies a basically unstable situation, because at any time the infection may be reintroduced by carriers or vectors from the outside.	[12]

**1963**	The word elimination is used according to its derivation from the Latin ex and limen - beyond a threshold. Since a threshold is involved, this is not a final process and the threshold specified may vary from disease to disease. In general, the agent may be permitted to persist as long as it does not - or only vary rarely - cause human disease. Alternatively the threshold may be the boundaries of a defined geographic area.	[32]

**1982**	Elimination is the disappearance of transmission of an infection from a small or large area, with a country or a continent ultimately becoming free from infection. Though reversible by importation of infection from other areas, the achievement of elimination, even if temporary, is important because it demonstrates the feasibility of ultimate eradication throughout the world.	[34]

**1984**	Regional elimination is the complete cessation of indigenous transmission in a defined geographic area, with the implication that, depending on frequency of importations and ease with which they can be contained, certain control measures can be modified or dropped.	[35]

**1993**	Refers to cessation of transmission of a disease in a single country, continent, or other limited geographic area, rather than global eradication (e.g., polio in the Americas). It is also theoretically possible to "eliminate" a disease in humans while the microbe remains at large (e.g., neonatal tetanus). Although a disease itself may remain, a particularly undesirable clinical manifestation of it may be prevented entirely (e.g., blindness from trachoma) or new transmission interrupted (e.g., infectious yaws). Control of a disease or its manifestations to a level that it is no longer considered "a public health problem," as an arbitrarily defined qualitative (e.g., onchocerciasis in West Africa) or quantitative (e.g., leprosy incidence below one case per 10,000 population) level of disease control.	[18]

**1998**	Reduction to zero of the incidence of infection caused by a specific agent in a defined geographic area as a result of deliberate efforts; continued measures to prevent reestablishment of transmission are required.	[13]

**2006**	Nationwide per year fewer than three 'epidemiologically linked' cases of malaria infection without an identifiable risk factor other than local mosquito transmission, for three consecutive years.	[48]

**2008**	Interrupting local mosquito-borne malaria transmission in a defined geographical area, i.e. zero incidence of locally contracted cases, although imported cases will continue to occur. Continued intervention measures are required.	[46]

Both of Payne's alternatives for elimination - that it could refer either to a) a reduction of transmission below a threshold, or b) a reduction within a defined geographic area - appeared elsewhere in published literature. In 1962, Hinman posited that there were four separate states that could occur: control, elimination, regional eradication, and eradication [33]. Under his definitions, control "*leaves the occurrence of the disease at a reduced, but presumably acceptable, level*," while elimination means that "*the disease no longer occurs on a continuing basis in the area, but the threat of reintroduction of disease from outside this area...is so great that continuing control efforts are required*." As a final stepping stone on the way to global (or what he called "true") eradication, regional eradication is achieved when "*it would not be necessary to pursue actively the control measures; surveillance and prompt response to importation are capable of maintaining the area free of disease*."

However, Payne's second definition is the one that would make its way into modern usage: the first official codification of "elimination" as a distinct concept from "eradication" appears to have been the 1982 Report on the International Conference on the Eradication of Infectious Diseases [34]. That report maintained a definition of eradication consistent with past usage, but explicitly described the concept of elimination in terms of geographic scope:

*"Elimination is the disappearance of transmission of an infection from a small or large area, with a country or a continent ultimately becoming free from infection. Though reversible by importation of infection from other areas, the achievement of elimination, even if temporary, is important because it demonstrates the feasibility of ultimate eradication throughout the world." *[34]

The fragile nature of elimination captured by this definition - that it was a reversible, possibly fleeting reprieve from the burdens of malaria, perhaps ultimately as tenuous as the "regional eradication" dismissed by Soper, Cockburn, and Fenner - was wrestled with by later authors.

"*Regional elimination is the complete cessation of indigenous transmission in a defined geographic area*," wrote Chin in reference to poliomyelitis, "*With the implication that, depending on frequency of importations and ease with which they can be contained, certain control measures can be modified or dropped*" [35], but this last clause was explicitly disavowed by other authors. For example, the definitions that generally remain accepted today were expounded by Dowdle *et al *in 1998 [13]. They defined elimination as a reduction to zero within a defined geographic area, but cautioned that "*continued measures to prevent reestablishment of transmission are required*."

Malaria programmes gradually returned to an objective of control rather than elimination as the GMEP approach proved unfeasible in a number of contexts. The original GMEP discussions had defined control as an alternative to the time-limited goal of eradication; for example, the WHO Expert Committee defined control as "*the reduction of the disease to a prevalence where it is no longer a major public health problem; the concept carries the implication that the programme will be unending, control having to be maintained by continuous active work*" [36]. As early as 1961, the WHO had come to terms with the fact that a one-size-fits-all "eradication" approach was unlikely to succeed everywhere, and allowed that an extended pre-eradication period would be required in those countries not yet ready to embark on an eradication program [37]. Over time, it was accepted that essentially indefinite pre-eradication programs "*which cannot move to eradication programmes within the foreseeable future are more in the nature of control programmes*" [38]. "*That the word 'control' should now be heard within the 'palace walls' is certainly a sign of changing philosophies and times*," de Meillon wrote in 1969 [39].

The 15^th ^report defined control as "*an organized effort to carry out those antimalaria measures that are possible with the available resources and suitable under the prevailing epidemiological conditions, with the objective of achieving the greatest possible reduction of mortality and morbidity.*" Officially, both the 15^th ^and 16^th ^WHO Expert Committee Reports of the early 1970s continued to maintain that eradication remained the true goal of all malaria programs, with control not considered a formal endpoint, but rather an operational stage en route to the ultimate goal of ending transmission. However, the pessimism of subsequent accounts [40-42] confirm the general perception that control was in fact an "alternative target" [42] that represented an endpoint unto itself.

### Elimination today

"*Malaria elimination...does not require...a complete absence of reported malaria cases in the country*," state 2007 WHO guidelines. "*Imported malaria cases will continue to be detected due to international travel, and may on occasion lead to the occurrence of introduced cases in which the infection is a first generation of local transmission subsequent to an imported case*" [5]. This distinction reflects a careful categorization made during GMEP of different degrees of local transmission resulting from imported cases. Malaria was defined as "autochthonous" when contracted locally within a region of interest, but this local transmission was subdivided into "introduced" cases, or those "*directly secondary to a known imported case - i.e. the first step only of renewed local transmission*" [43], and "indigenous" cases, or those resulting from further degrees of transmission [44]. GMEP sought to decrease to zero the number of indigenous cases [37], while recognizing that introduced cases were inevitable [36]. "*An important implication*," described in the context of measles elimination, "*is that a disease can be eliminated as an indigenous problem even though there are recurrent outbreaks, a few of which might be quite large or involve a number of generations of cases*" [45].

In contrast, many contemporary definitions state that any local transmission - which presumably includes introduced cases - is unacceptable. Current understanding of elimination holds that it involves "*the interruption of local mosquito-borne malaria transmission in a defined geographical area, creating a zero incidence of locally contracted cases*" [7,46], with the caveat, "*imported cases will continue to occur and continued intervention measures are required*." The WHO's guide, "Malaria Elimination: A field manual for low and moderate endemic countries" supports this definition, stating, "*The goal of the elimination programme is to halt local transmission area- or countrywide, clear up malaria foci, and reduce the number of locally acquired cases to zero*," and later, "*When a country has zero locally acquired malaria cases for at least three consecutive years, it can request WHO to certify its malaria-free status*" [5]. Similarly, an official statement of "the WHO perspective," defines elimination as "*0 incidence of locally contracted cases*" [2].

This discrepancy exactly mirrors the inexactness in definitions that occurred during GMEP. "*In a small number of programmes*," Yuketiel wrote in 1960,

*"The statement, 'reduction of the number of cases to zero', was taken literally and spraying operations were unnecessarily prolonged. The principle is, however, an epidemiological one, and its basic meaning is that cases should be so low in number that either they could not per se re-establish transmission under the prevailing entomological conditions, or, more important, they can be detected and treated in time in the course of surveillance operations with the same result." *[47]

The 2006 WHO report "Informal consultation on malaria elimination" directly highlights this conflict, pointing out that defining elimination as a reduction to zero incidence "*does not clearly take into account the probable persistence of the incidence of disease due to the presence of imported cases*" [48]. It continues:

*"A complete absence of locally acquired malaria cases is epidemiologically unlikely: as long as *Anopheles *mosquitoes are present and in contact with the population, occasional infection of local mosquitoes by gametocyte carriers that visit or pass through a country cannot be prevented. Occasional first-generation, locally acquired infections (introduced cases) thus continue to occur, except in areas where the density and survival of *Anopheles *mosquitoes are systematically reduced by diligent vector control measures to a level where transmission is no longer likely." *[48]

Conceptualizing elimination as a "*reduction of case transmission to a predetermined very low level*" [49], but not necessarily zero, is embodied in the WHO's 2007 requirements for elimination certification. To be certified as malaria-free, the WHO requires that a country have an "*absence of clusters of three or more epidemiologically-linked autochthonous malaria cases due to local mosquito-borne transmission, nationwide for three consecutive years*" [50]. Preconditions for proving this interruption of transmission beyond a "reasonable doubt" include strong surveillance mechanisms, case reporting, diagnosis, and follow-up of cases. As such, having some low level of local transmission does not mean that a country has failed to maintain elimination. The WHO correspondingly defines elimination for other diseases as reductions below defined thresholds rather than the complete absence of disease within a defined area. For example, tuberculosis elimination is defined as less than one case per million people, leprosy elimination is defined as less than one case per ten thousand, and the onchocerciasis elimination programme in Africa seeks only to decrease incidence to the point that it is not "of public health and socioeconomic importance" [48].

Similarly, re-establishment of transmission is considered to occur when "*more than two epidemiologically linked cases of malaria infection per year without an identifiable risk factor other than local mosquito transmission*" occur "*in the same geographical focus, for two consecutive years for *P. falciparum *and for three consecutive years for *P. vivax" [48]. This threshold of three cases in two consecutive years is in the spirit of GMEP guidance that "*some minute foci should not lead to the deletion of the area concerned from the eradication register, provided that an endemic state is neither re-established nor appears to be re-established*" [51], but the significance of a threshold of three as compared to a larger number of incidental cases or a population-based rate is not justified.

Nevertheless, general perception of "elimination" as "*reduction to zero of the incidence of infection*" [6] persists. The incongruity between a goal of absolute zero and the allowance that at least some locally-acquired infections are inevitable has important operational consequences. Seeking to prevent every autochthonous case may require more stringent operational requirements than permitting a low level of local transmission, since marginal returns for key interventions will likely decrease at high coverage levels [52]. Additionally, expenditure required to maintain absolute zero may be substantially greater than that if some transmission is allowed [53], although the magnitude of this effect may vary by intervention type and context [52]. For example, a recent analysis of the feasibility of elimination in Zanzibar assessed operational and financial requirements for preventing all locally-acquired infections, resulting in the forecasting of extremely onerous operational requirements to achieve elimination with costs that would be significantly higher than those of a control program for the foreseeable future [54]. Indeed, one of the strongest arguments against elimination of any disease involves the increasing costs associated with finding and treating decreasing numbers of cases [55,56], since the final few cases may require an enormous outlay of resources that may be considered disproportionate to the harm averted. If a different definition of malaria elimination is assumed that allows for occasional local transmission, these requirements may be significantly less burdensome; in certain situations, this shift in definition may determine the perceived feasibility of elimination.

Current definitions that stipulate elimination involves both a reduction to zero and a requirement that sufficient control measures are maintained to prevent onward transmission from imported cases seem to conflate two distinct states described historically as regional eradication and elimination, producing a new one that may prove somewhat paradoxical. Payne's 1963 definition describes that elimination can be conceived of as reduction of disease below any given threshold [32], producing a state that is a concept distinct from "regional eradication" [33]. Such a state is necessary because of the recognition that some level of local transmission is inevitable as long as importation persists; once a sufficiently large region achieves transmission below the elimination threshold, then dramatic importation reductions may make regional eradication feasible. A state of absolute zero elimination combines the absence of transmission that occurs under regional eradication with the constant importation that occurs under a transmission threshold conception of elimination. The improbability of such a state can be described quantitatively.

### A quantitative description of elimination and importation

The difference between global eradication, elimination, and control is the difference between absolute zero, nearly zero, and low. Elimination in a specific region implies that endemic transmission - that is, indigenous incidence of malaria infection that would persist even if all importation were halted - has been interrupted [57], but until global eradication has been achieved, every region will import malaria, and in areas where malaria was formerly endemic, there is also likely to be some onwards transmission from those cases. While this conceptual distinction - that regional elimination will not imply the complete absence of malaria, but rather a lack of endemic transmission - is simple, it is difficult to define operationally. How should endemic transmission be distinguished from a high rate of malaria importation? Is it sensible to declare a state of elimination if malaria is frequently imported? Or if those imported cases frequently lead to introduced cases? In some ways, each one of these questions is referring to the same definitional problem of where to draw a line along a continuum that is best described quantitatively.

The relevant quantitative concepts for transmission are described in WHO documents as "receptivity," "vulnerability," and "malariogenic potential." Receptivity is described as "*the abundant presence of anopheline vectors and the existence of other ecological and climatic factors favouring malaria transmission*" [5], a concept that corresponds to the definition of reproductive numbers. The basic reproductive number, denoted R_0_, is defined as the expected number of human cases that would arise from a single introduced malaria case in a population with no immunity and no control. Most places where malaria has been eliminated have at least some degree of outbreak control in the form of medical attention and outbreak investigations, so the appropriate measure of receptivity is called the reproductive number under control, R_C _[58].

Vulnerability has been defined as "*either proximity to malarious areas or...the frequent influx of infected individuals or groups and/or infective anophelines*" [59]. A more precise definition is the malaria importation rate, which for some well-defined region sums up all of the infections that can be traced outside of the region in the previous parasite generation, and is described as number of imported human malaria cases per 1,000 population per year. Malariogenic potential is defined as "*the interaction and effects of receptivity and vulnerability*," and "*can be considered as proportional to the amount of infection imported (vulnerability) and as an exponential function of the estimated degree of receptivity (density and species of vectors, climatic factors, etc.)*" [51]. If quantified as the product of receptivity and vulnerability, it measures the number of introduced malaria cases.

A minimal requirement for malaria elimination is that R_C _< 1 [57], or else malaria would tend to become endemic again. Each imported malaria case is expected to generate R_C _new cases, and each one of those cases would also generate R_C _cases, and so on. This naturally stochastic process can be modeled as a branching process that describes the probability distribution function of the number of cases that are expected from each imported malaria case for a given R_C_. The expected number of locally-acquired cases that can be traced back to each imported case is R_C _in the first generation, R_C_^2 ^in the second, and R_C_^n ^in the nth. When R_C _< 1, the limit of this series as n goes to infinity is R_C_/(1-R_C_). Although R_C _is challenging to measure entomologically at low transmission intensity, this equation provides a simple way of estimating it from the observed numbers of imported and secondary cases. Assuming strong surveillance, imported cases may be distinguished from locally-acquired infections during outbreak investigations by their recent travel history to an endemic region. The ratio of locally-acquired to imported cases is then approximately indicative of the current level of R_C_. For example, the branching process equation indicates that a ratio of 1 locally-acquired case to 1 imported case would be expected if R_C _= 0.5, while a ratio of 9:1 (i.e., nine locally-acquired cases per imported case) would occur if R_C _= 0.9, and 1:3 (i.e., one locally-acquired case for every three imported cases) would be observed when R_C _= 0.25. This ratio provides a measure of progress towards elimination in areas where imported malaria is frequent, although its precision will depend upon the degree to which it is possible to classify cases accurately as imported or locally-acquired.

**Figure 1:**
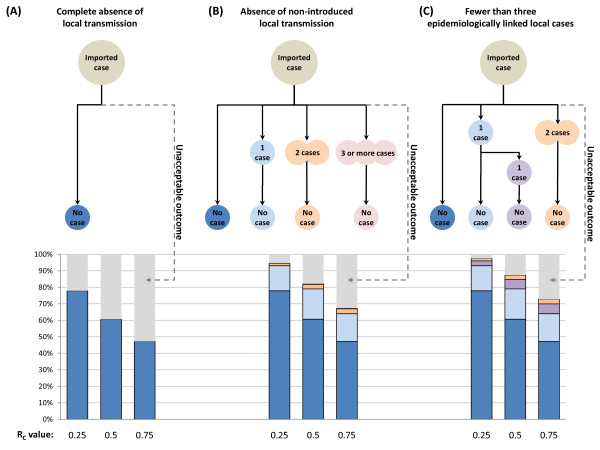
**Branching process diagrams for three definitions of elimination**. The Poisson probability of each branch permitted under three elimination definitions and the total probability of all other unacceptable outcomes are depicted for three R_C _values.

**Figure 2:**
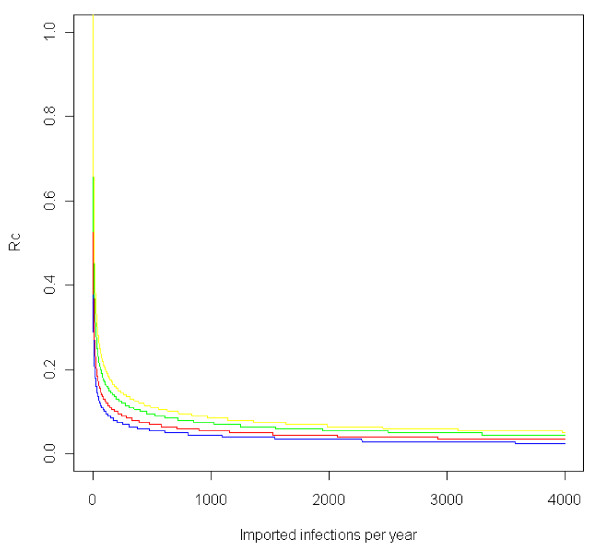
**Maximum R_C _at which the probability of failing to meet elimination criteria of fewer than three epidemiologically-linked local cases in three consecutive years is kept below risk thresholds**. Thresholds of 1% (blue), 5% (red), 25% (green), or 50% (yellow) risk of failure are depicted, assuming the number of cases resulting from each case follows a Poisson distribution with mean = variance = R_C_.

It is, in fact, quite difficult to enumerate all of the possible outcomes from a single imported malaria case, but it is reasonably simple to compare the likely outcomes permitted within a specific operational definition of elimination, such as the WHO requirement of fewer than three epidemiologically linked cases occurring in three consecutive years (Figure 1). Using the branching process model depicted in Figure 1C and assuming a Poisson distribution, the probability of three or more cases occurring given a single importation event was calculated for a range of importation levels and R_C _values. Given the probability of this "failure" in one year, *p*_*1*_, the probability of having at least one year with three or more cases over the course of *n *years with *i *importation events per year can then be calculated as:

In any place where R_C _> 0, there is some risk of losing "malaria-free" status from each imported malaria case, simply by chance. For example, Figure 1A depicts the probability of any local transmission occurring from a single importation event. Even if R_C _is reduced to 0.25, there remains a greater than 20% probability of at least one onwards transmission event occurring. The more cases are imported, the greater the chance of having a cluster of three or more cases. Model results indicate that R_C _must be very small or that importation must be reduced to extremely low levels before the probability of losing malaria-free status is reduced to acceptably low levels (Figure 2).

It is thus clear that elimination as generally understood, meaning "zero local transmission," is an essentially impossible goal for every country, including countries like the United States where there are approximately 1,200 importation events per year [60]. These imported infections resulted in three locally acquired cases in Virginia in 2002 and eight in Florida in 2003, for a total of 63 domestic outbreaks with a total of 156 locally-acquired malaria cases between 1957 and 2003 [30]. Such sporadic transmission belies any definition of the term equating elimination to a complete lack of local transmission. According to the branching process pictured in Figure 1C, maintaining less than a 25% chance of having three or more locally transmitted cases in a single year given the amount of importation into the U.S. would require an R_C _of about 0.04. Such extraordinarily low transmission potential is only achievable if nearly every case is sequestered from mosquitoes or rapidly treated; achieving sufficient reductions in contexts with much higher intrinsic transmission potential would thus be practically impossible at present.

### New provisional definitions

The historical review highlights a set of terms and concepts that have been variously referred to as control, elimination, and eradication. To set achievable goals and feasible strategic plans, it is necessary to resolve the definitional ambiguities surrounding these terms (with the exception of eradication, which is a global rather than national ambition). In formulating new definitions that will be applicable to all endemic countries, it is first essential that the conceptual states represented by each term are clearly and unequivocally described. Conceptual definitions are qualitative descriptions of the epidemiological states that a country or region can achieve through diverse control measures, treatment, and health system strengthening. Those proposed here could be applied to any subset of the human *Plasmodium *species.

A conceptual definition is not useful, however, until its operational implications for a specific context are clearly defined in terms of quantitative goals and metrics. Good operational definitions to guide malaria programs have clear relationships with their underlying concepts, provide realistic milestones that are technically possible to achieve, and convey accurate information about relative and absolute progress towards goals based on direct measures of malaria. Unlike conceptual definitions, appropriate operational definitions may not be universally applicable; for example, GMEP's precise operational guidance designed to interrupt malaria transmission in places like Western Europe and the Americas failed to prove relevant to the far different socio-epidemiological context of Africa [51,61]. Additionally, operational definitions need to take into account the unique epidemiological and biological characteristics of each individual parasite species. For example, operational definitions that apply to *P. vivax *must take into account the presence of dormant liver-stage infections and their propensity to relapse. While it is outside the scope of this paper to derive comprehensive operational definitions, some metrics that will facilitate operationalization of the provisional conceptual definitions proposed are suggested here.

Reducing transmission of malaria from highly endemic levels remains the immediate task in much of the world. In cases where elimination is deemed technically, operationally, or financially unfeasible, countries may seek to reduce malaria to very low levels without achieving interruption of endemic transmission. Such an achievement, which generally has been encompassed by the imprecise term "control," is here called "controlled low-endemic malaria":

**Controlled low-endemic malaria **refers to a state where interventions have reduced endemic malaria transmission to such low levels that it does not constitute a major public health burden, but at which transmission would continue to occur even in the absence of importation.

Since the "controlled" component of this definition would not apply to a region in which malaria transmission intrinsically occurs at such a low level, such a setting would thus be described simply as "low-endemic malaria."

This definition is conceptual, and identification of a specific, meaningful threshold, such as an upper limit on prevalence, will be required to make it operationally useful. Future investigations are needed to understand how realistic and verifiable thresholds consistent with this conceptual definition should be defined in specific contexts, given the geographical variation and seasonality in baseline endemicity. Guidance may be derived from field observations; for example, previous investigations have demonstrated that areas in Africa that have achieved very low prevalence rates (e.g., less than 1%) manifest comparatively low specific mortality and severe disease outcomes, with malaria's contribution to all-cause childhood mortality significantly reduced [62]. Similar patterns apply to the patterns of malaria endemicity versus malaria morbidity [63]. These observations suggest a good starting point for discussions about operational definitions of controlled low-endemic malaria might involve a state where interventions have reduced the average parasite rate in a nationally representative sample below 1% prevalence during the peak transmission season (an arbitrarily defined but clinically meaningful threshold), while prevalence levels in subpopulations remain below a higher threshold (e.g., lower than 5% prevalence) to allow for heterogeneity in endemicity caused by focal transmission. Alternatively, clinical metrics, like a positive fraction of tested fevers below 5%, might be considered if strong surveillance sites are available with consistent patient populations and testing rates.

Achieving controlled low-endemic malaria in a region with a naturally high level of endemicity is a significant accomplishment, and it represents a logical end-state for many malaria programs over the near term [3]. Alternatively, programmes may aim to reduce transmission even further, with the goal of eventually getting rid of malaria altogether. Previous definitions have struggled, however, to conceptualize the middle ground between controlled low-endemic malaria and absolute zero. It is clear that one of the chief distinctions between controlled low-endemic malaria and elimination must involve the interruption of endemic transmission so that malaria transmission would cease if importation were halted. Additionally, all modern definitions of the term [5,7,9] indicate that elimination means malaria is nearly always absent from the region. In other words, elimination also implies that malaria has been reduced below a threshold.

**Figure 3:**
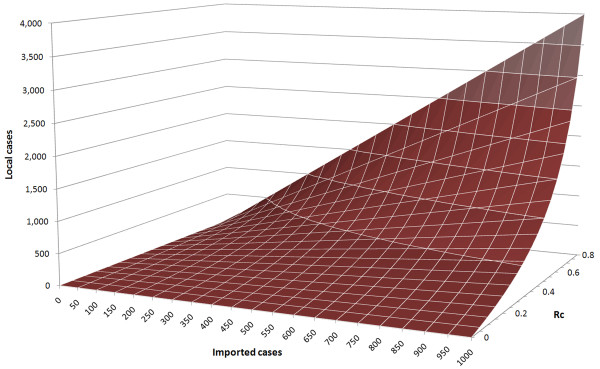
**Approximate number of locally-acquired cases (both introduced and indigenous) expected to result from a given number of imported cases under a particular level of R**_**C**_.

Operational threshold criteria such as those currently proposed by WHO - that elimination is achieved when there are no more than three linked cases in three consecutive years [48] - are nearly impossible to achieve for a country with a high malariogenic potential, even if local infectious reservoirs have been eliminated and endemic transmission has been interrupted, unless R_C _is lowered to extraordinarily low levels (Figure 2). A hypothetical country that had achieved R_C _= 0.25 and successfully interrupted endemic transmission yet still imported 1,000 malaria cases a year could expect to see over 300 local cases each year resulting from those importations (Figure 3); elimination by a definition that requires virtually no malaria is thus essentially impossible in high importation contexts like much of sub-Saharan Africa at present. Even in an island region like Zanzibar, where annual importation from endemic neighbours could theoretically range from 1,000 to nearly 13,000 [64], it is clear that such stringent standards are operationally infeasible.

To disambiguate the previous concepts and establish milestones for countries that want to measure progress towards elimination, two conceptual definitions are proposed for this middle ground:

**Controlled non-endemic malaria **refers to a state where interventions have interrupted endemic transmission and sharply limited onward transmission from imported infections, but where high malariogenic potential means that some level of local transmission is inevitable; elimination would naturally follow if all malaria resulting from imported infections could be prevented.

**Elimination **refers to a state where interventions have interrupted endemic transmission and limited onward transmission from imported infections below a threshold at which risk of reestablishment is minimized. Both capacity and commitment to sustain this state indefinitely are required.

Achievement of controlled non-endemic malaria may essentially equate to elimination in countries with very low intrinsic transmission potential, while in areas with high potential, imported cases likely will lead to sufficient onward transmission that malaria may not be considered to have been eliminated. It is possible for countries to move from controlled non-endemic transmission to the very low levels of malaria that would satisfy the definition of elimination. In some cases, all that would be required is to lower the number of imported malaria cases. The second part of the elimination definition is also critical; countries that are recognized to be malaria-free have made the commitment to sustain elimination, and they have demonstrated that they are capable of doing so. Such demonstration includes surveillance and health systems that are strong enough to convince a skeptical observer that endemic transmission is not occurring anywhere within the country. Otherwise, controlled non-endemic malaria describes an entire spectrum between controlled low-endemic malaria and elimination. It should be noted that, in the case of *P. vivax*, achieving the interruption of endemic transmission required to meet these definitions will require preventing transmission from relapsing cases; longer timeframes or different operational strategies thus may be required.

Operational definitions of either controlled non-endemic malaria or elimination should establish a set of metrics to verify the absence of endemic transmission. A sufficient *a priori *definition of non-endemic malaria is that R_C _< 1, so that every introduced malaria case will deterministically go extinct, though for interruption on reasonable timelines, R_C _will need to be < 0.5 [65]. The ratio of locally-acquired to imported cases provides a rough way of operationally estimating R_C _to assess the degree to which transmission remains endemic versus non-endemic, even if the rates of malaria importation remain too high to relax vector control measures and rely on health systems. A suitable definition for controlled non-endemic malaria is that the ratio of locally-acquired to imported cases is less than 1:1. Additional metrics might include serology surveys in which no children <2 test positive for antibodies; other guidance has been suggested for diseases like measles [66].

Operational definitions differentiating elimination from controlled non-endemic malaria must establish acceptable transmission thresholds that take importation levels into account. A specific number of local cases, as used in the WHO definition [48], may be suitable for one region but infeasible or overly lax for another, depending upon the malariogenic potential (Figure 3). One context-specific way to set such a threshold would be to identify the number of indigenous cases that surveillance and response capacity can manage operationally with extremely low risk. For example, perhaps a country's vigilance is sufficient to respond to approximately one transmission chain resulting from importation per week, or 50 per year. If 1,000 cases were imported into that country annually, there would be some number of introduced cases and, occasionally, indigenous transmission from those introduced cases. The branching process models applied here can be used to demonstrate that there would be approximately 200 introduced cases and 50 indigenous cases if R_C _were about 0.2, or in operational terms, robust surveillance identifies a total of approximately 0.2/(1-0.2) = 0.25 locally-acquired cases per imported case for an annual ratio of one locally-acquired case to every four imported cases. Below this threshold, risk of reestablishment of malaria could be deemed acceptably low; a higher rate of importation would require a lower ratio to ensure this same level of risk. More stringent thresholds could also be suggested below this level, with the most extreme being a requirement of zero positive tests among febrile patients with no travel history. These quantities may change over time and can be used as feedback to revise programmatic goals in subsequent planning cycles. Further evaluation and refinement of these sorts of operational metrics will be required.

## Conclusions

A renewed commitment to eradicating malaria has revitalized debate about the proper goals and priorities of malaria programs, international agencies, and the donor community. An essential component of these discussions is a clear understanding of how these goals are to be defined using standard malaria metrics, but the old terms control and elimination have often been adopted without clarification of their conceptual and operational meanings. Here, new definitions have been proposed to refine the previous concepts in a way that is easily operationalized.

The definition for controlled low-endemic malaria accepts historical guidance that the goal of control should be to reduce malaria to levels at which it no longer poses a public health problem [36], but it goes further in requiring that such a concept be operationalized; it was suggested here that this conceptual state may be achieved through interventions that lower the prevalence of malaria to below a specified threshold, like 1% prevalence. Although available evidence suggests that mortality and morbidity are likely to be substantially reduced at such a low levels of endemicity, careful derivation of operationally meaningful thresholds in different eco-epidemiological contexts is needed. Having quantitative targets of this nature will allow malaria programmes to measure progress towards specific targets and plan resources and strategy appropriately.

After achieving controlled low-endemic malaria, many countries may consider malaria elimination, ideally through a detailed malaria elimination feasibility assessment [54]. The collective actions of countries pursuing elimination could eventually lead to global malaria eradication. To this end, this review has sought to clarify the concept of elimination and explicitly describe the differences between it and the alternative of controlled low-endemic malaria. The challenge of applying previous definitions of elimination became apparent during an assessment of the feasibility of elimination in Zanzibar [54], which found that eliminating the final few cases required to achieve zero local transmission required a prohibitive outlay of resources, while achieving a goal of eliminating endemic transmission but permitting some small amount of local transmission to occur as a result of imported cases was far more operationally and financially feasible. Countries currently attempting elimination must not hold themselves to a more rigorous standard than do the U.S. and Europe, which experience occasional local transmission.

The newly defined state of controlled non-endemic malaria establishes an important milestone that preserves a definition of elimination in line with modern conception of that state as a general absence of malaria, while recognizing that regions with very high importation and intrinsic transmission potential require a more operationally feasible goal beyond the achievement of controlled low endemicity. Such historically high-burden countries can use this milestone to demonstrate the capacity to interrupt endemic transmission and take a step towards eventual elimination as importation from neighbors is reduced and transmission potential is even further limited.

National malaria programmes must set well-defined goals to mark progress, but the wide variety of transmission contexts across and within countries suggests the need for flexibility in operationalizing these conceptual definitions. The simple branching processes described here demonstrate the infeasibility of achieving or maintaining zero local transmission for any country with high intrinsic transmission potential to which substantive importation of infections continues to occur. Although an elimination threshold of fewer than three cases per year may be attainable for an area of naturally low endemicity like Western Europe, it is not plausible in regions of much higher endemicity and importation. Useful definitions of states like control and elimination must therefore be offered not only on a universal conceptual level, but also on an operational one that accounts for local context. For example, adopting a threshold that involves a rate of locally-acquired cases per 1,000 population rather than a fixed number of cases would more readily allow that threshold to be applied to regions of different population sizes. In lieu of a specific number, a ratio was suggested here to measure not the absolute amount of malaria (which will vary based on importation) but rather the relative amount of locally-acquired versus imported cases. Operational definitions according to robust metrics will allow malaria programs to set achievable, if challenging, goals.

Attempts to achieve and maintain absolute zero in high transmission regions will result in enormous outlays of resources in quixotic quests for impossible goals. In some areas of sub-Saharan Africa, GMEP authors' arguments against "regional eradication" remain valid today as a way of illustrating why no country can go it alone. The states of controlled low-endemic and non-endemic malaria represent important goals that countries can aim to attain, even with high rates of malaria importation. Elimination can, in theory, provide an operationally feasible goal for the countries at the center of regional initiatives, while controlled non-endemic malaria is a more appropriate aim for peripheral countries that border on highly endemic countries. Controlled low- or non-endemic malaria can be reinforced by control in neighbouring countries or other efforts that expand the extent of regional initiatives until regional elimination is achieved. In other words, if programmes are to have attainable goals, it must be accepted that "absolute zero" on a local scale is highly unlikely until regional or continental elimination or global eradication becomes a reality.

## Competing interests

The authors declare that they have no competing interests.

## Authors' contributions

BM and DLS conceived of this review. JMC carried out the review and drafted the manuscript, and JMC and DLS conducted the quantitative analysis. RWS and BM participated in the interpretation and presentation. All authors read and approved the final manuscript.
